# Design improvement of the conversion mechanism from balloon inflation to bending motion for inflatable film actuators

**DOI:** 10.1038/s41378-023-00526-y

**Published:** 2023-05-09

**Authors:** Y. Hori, S. Konishi

**Affiliations:** 1 Graduate School of Science and Engineering, Ritsumeikan University, Shiga, Japan; 2 Department of Mechanical Engineering, Ritsumeikan University, Shiga, Japan; 3 Ritsumeikan Advanced Research Academy, Kyoto, Japan; 4 Ritsumeikan Global Innovation Research Organization, Kyoto, Japan

**Keywords:** Engineering, Physics

## Abstract

Various soft actuators have been investigated to overcome the drawbacks of conventional solid machines and explore the applications of soft robotics. In particular, and because they are expected to be applicable in minimally invasive medicine because of their safety, soft inflatable microactuators using an actuation conversion mechanism from balloon inflation to bending motion have been proposed for high-output bending motion. These microactuators could be applied to create an operation space by safely moving organs and tissues; however, the conversion efficiency could be further improved. This study aimed to improve conversion efficiency by investigating the design of the conversion mechanism. The contact conditions between the inflated balloon and conversion film were examined to improve the contact area for force transmission, with the contact area dependent on the length of the contact arc between the balloon and force conversion mechanism and on the amount of balloon deformation. In addition, surface contact friction between the balloon and film, which affects actuator performance, was also investigated. The generated force of the improved device is 1.21 N at 80 kPa when it bends 10 mm, which is 2.2 times the generated force of the previous design. This improved soft inflatable microactuator is expected to assist in performing operations in a limited space, such as in endoscopic or laparoscopic operations.

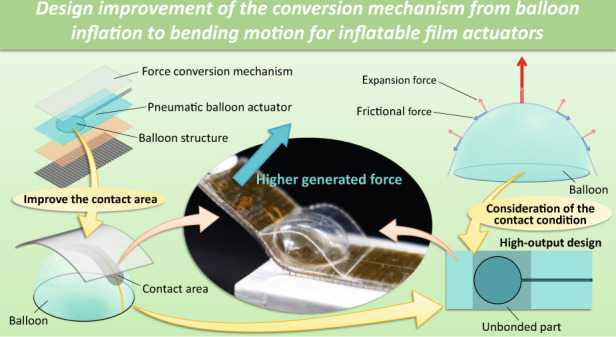

## Introduction

Conventional robots constructed using hard materials have advantages in accuracy, stability, and modeling^1,2^. In recent years, soft robots have been explored to solve problems that are difficult for rigid systems and to broaden the possibilities of the robotics field^1–11^. Soft robots, constructed using soft and flexible materials, exhibit adaptive shape deformation when making contact with objects, making them inherently safer to use in situations where contact with humans and fragile objects occurs. As a result, soft surgical robots have been reviewed and discussed in terms of engineering design, fabrication techniques, and human–robot interactions^3^.

**Fig. 1:**
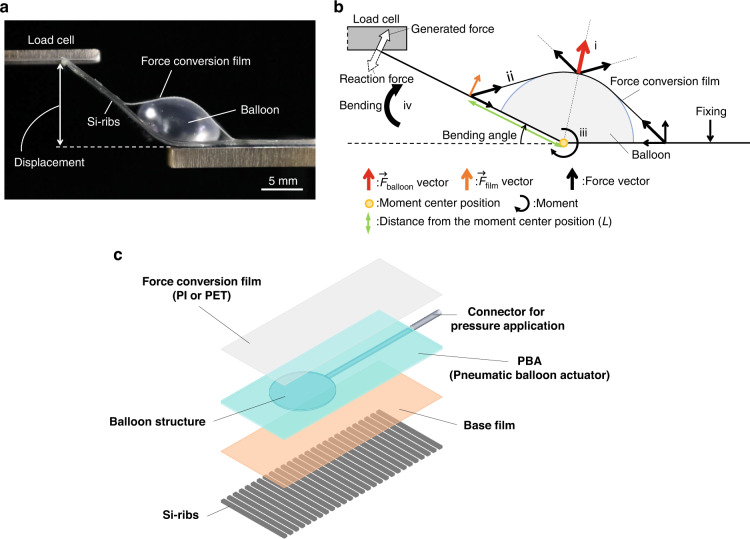
Appearance and structure of the inflatable film actuator and driving principle. **a** Appearance of the actuator. **b** Mechanical model representing the driving principle. **c** Structure of the actuator. In previous studies^16,17^, polyimide (PI) film was used as the force conversion film; however, in this study, polyethylene terephthalate (PET) film was used as the force conversion film. PET is a more elastic material than PI, and by improving the method of bonding with PBA, the generated force increased approximately 1.4 times (Fig. 7a Conventional)

In previous studies, we developed soft actuators using pneumatic balloons, collectively named pneumatic balloon actuators (PBAs), and reported bending, torsion, and contraction motions^12–17^. The PBAs were fabricated using MEMS technology, including lithography, etching to form a casting mold, and molding of soft materials. Silicone rubber materials, such as polydimethylsiloxane (PDMS), are often used as soft and elastic materials. PBAs comprise two thin elastic films with different mechanical characteristics and utilize the asymmetrical deformation of these two elastic thin films for bending and twisting motions. In parallel, a conversion mechanism has been proposed for the high-output bending and contracting motion of soft inflatable microactuators^14,16,17^. In these microactuators, the force conversion mechanism effectively converts the expansion force and displacement of the inflatable balloon into the desired motions with high force density. Figure 1a shows the appearance of the bending actuator with the conversion mechanism. As shown in Fig. 1c, the actuator consists of a PBA inserted between a force conversion film on the upper layer and Si-ribs on the lower layer. The force conversion film efficiently converts the balloon’s expansion force into a bending force, and previous work has succeeded in significantly improving the generated force^16,17^.

According to numerous studies, the desired actuations have been realized using force conversion mechanisms at the microscale^18–22^. The actuator output, which decreases with miniaturization, has been converted and amplified by the force conversion mechanism to ensure its effective utilization in various actuations. The micro pneumatic gripper has been actuated by pressure, which has been converted into a reciprocating motion using piston bellows^18^. The flexural hinges, which have been found to convert the reciprocating motion into a grasping motion, have been fabricated by RIE and SU-8 UV-deep lithography and have been found to exhibit different properties depending on the material used. Microtweezers have converted the thin membrane expansion force due to applied pressure into a cantilever displacement for grasping motion^19^. In addition, the gripping force can be verified from the strain of the cantilever. The uniflex microactuator has been found to amplify tiny strains of the piezoelectric element, converting them into a large displacement^20^. Strain amplification has been realized by combining a unimorph with a strain amplification mechanism for flexural motion. An actuator that converted and amplified piezoelectric in-plane forces into out-of-plane forces and out-of-plane displacements has also been reported^21^. This actuator was developed by combining piezoelectric in-plane actuators and MEMS-enabled scissor mechanisms. Two-axis actuators have converted in-plane motion into out-of-plane motion by utilizing the symmetry of the force conversion mechanisms^22^, with tilting motion utilizing the asymmetry of the force conversion mechanisms also reported.

In our previous work, we succeeded in increasing the output of this actuator by implementing the force conversion mechanism and achieved approximately 0.4 N (80 kPa) at a displacement of 10 mm (see Fig. 1a). Subsequently, by changing the material of the force conversion film from a polyimide film (PI) to a polyethylene terephthalate film (PET) and improving the bonding method, the adhesive properties were enhanced, with 0.55 N (force conversion film: PET, base film PI, 80 kPa) achieved under the same conditions. In the present work, we focused on the area of contact and attempted to further improve the force conversion efficiency. In this actuator, the balloon part expands when pressure is applied, and the force conversion film converts the expansion force into a bending motion. For this reason, as the contact area between the balloon and the force conversion film increases, the force conversion efficiency increases, and the generated force also increases. In this study, we further improved the generated forces using a high-efficiency design to increase the contact area between the balloon and the force conversion mechanism by means of a mechanical model.

### Design

#### Driving principle of inflatable film actuator

Figure 1a shows the appearance of the inflatable film actuator, and Fig. 1b shows the mechanical model of the driving principle for the actuator shown in Fig. 1a. The driving mechanism of the actuator is as follows:

i. The balloon expands based on the applied pressure, and the force conversion film is lifted upward. The force vector received by the film at this point is defined as the $\vec{F}$_balloon_.

ii. The $\vec{F}$_balloon_ is decomposed by the force conversion film and transferred in the forward and backward directions relative to the balloon by the tensioning of the conversion film.

iii. The component of the transferred force that is vertical to the PBA and ribs is designated $\vec{F}$_film_. A moment of force is generated that depends on $\vec{F}$_film_ and the distance *L* from the center of rotation of the actuator flexion, and the actuator starts bending.

iv. The actuator bends until the moment of force generated by the balloon inflation is counterbalanced by an equal and opposite moment exerted by the load at its tip. The generated force can be changed by controlling the applied pressure.

As the moment $\vec{M}$ that contributes to the bending of the actuator increases, the generated force of the actuator also increases, and $\vec{M}$ is calculated by the following equation:

1 $$\left|\vec{M}\right|=L\left|{\vec{F}}_{\text{film}}\right|\left(\propto \left|{\vec{F}}_{\text{balloon}}\right|\right)$$

As $\vec{{\boldsymbol{F}}}$_film_ increases in proportion to $\vec{{\boldsymbol{F}}}$_balloon_, the increase in the actuator moment depends on the three parameters, namely, $\vec{{\boldsymbol{F}}}$_film_, $\vec{{\boldsymbol{F}}}$_balloon_, and *L*. Therefore, we can use a mathematical model to generalize the trend of these parameters.

#### Formulation of three parameters ($\vec{{F}}$_balloon,_$\vec{{F}}$_film,_ L)

**Fig. 2:**
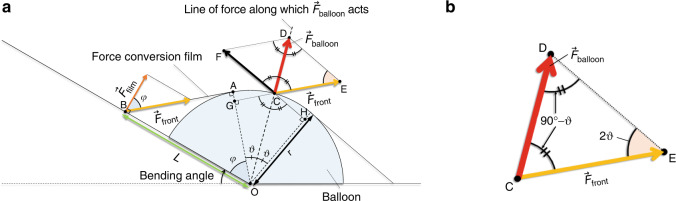
Geometric model for formulating ${\overrightarrow{F}}_{{\rm{balloon}}}$, ${\overrightarrow{F}}_{{\rm{film}}}$_,_*L*. O: center of rotation, A: edge point of contact between balloon and film, B: point at which $\vec{F}$_film_ acts in front of the balloon, D: tip of the $\vec{F}$_balloon_ vector, E: tip of the force $\vec{F}$_front_ that propagates to the front of the balloon from the decomposed $\vec{F}$_balloon_, F: tip of the vector decomposed on the opposite side of $\vec{F}$_front_. G: the intersection of OA and CE. H: points that are OC-axis symmetric with G. ∠AOB is *φ*, and ∠OAB is a right angle because the balloon and film are in contact at point A. *θ* is the contact angle between the line of force along which $\vec{F}$_balloon_ acts and the edge of contact between the force conversion film and balloon. **a** Overall view of the geometric model. **b** Enlarged view of ∆CDE

Figure 2a shows a geometric model for formulating the three parameters. In the model, we consider the drive at a displacement of 10 mm. The geometric model was prepared to extract the parameters for higher output from the structural features of the device and to efficiently improve the design. It was prepared not for precise analysis but for efficient derivation of high-output design by combining the determined tendencies with simple experiments. For ease of modeling, we assumed that the balloon was hemispherical. Although the actual balloon was not hemispherical, the hemispherical balloon model can be used to derive designs effectively and experimentally for enhancing the output by extracting the necessary parameters from the model and determining the tendencies, which is the aim of this study.

First, we calculate the length of the vector $\vec{F}$_front_, |CE| (see Fig. 2a), to obtain $\vec{F}$_film_. On the basis of trigonometry, since ∠DCE and ∠DCF are vertically opposite ∠OCG and ∠OCH, respectively, ∠DCE = ∠DCF = 90°− *θ*, we have ∠DCE = ∠CDE and ∠CED = 2*θ* because the quadrangle CEDF is a parallelogram. Focusing on △CDE, as shown in Fig. 2b, it is possible to obtain the following equation for |CE| using the law of sines:

2 $$\frac{\text{|CE|}}{\sin (90^\circ -\theta )}=\frac{\text{|CD|}}{\sin 2\theta }$$

3 $$\left|\text{CE}\right|=\frac{\text{|CD|}}{2\sin \theta }$$

Then, we can calculate the length of the vector $\vec{F}$_film_. $\vec{F}$_front_, which is transformed by the force conversion film and changes direction, is transmitted from position C–E to B–A through tension in the film. $\vec{F}$_film_, a component of the propagated $\vec{F}$_front_, is vertical to the OB. Thus, $\vec{F}$_film_ is calculated using the following equation:

4 $$\left|{\vec{F}}_{\text{film}}\right|={\text{|CE|}}\cos \varphi =\frac{\text{|CD|}}{2\sin \theta }\cos \varphi \,=\frac{\cos \varphi }{2\sin \theta }\left|{\vec{F}}_{\text{balloon}}\right|$$

The same model is used to generalize for *L*. As shown in Fig. 2a, L is the length of OB, and it is calculated using the curvature radius of the balloon *r* as follows:

5 $$L=\frac{r}{\cos \varphi }$$

From Eqs. (4) and (5), the moment $\vec{M}$ can be calculated using the following equation:

6 $$\left|\vec{M}\right|=\frac{r}{\cos \varphi }\frac{\cos \varphi }{2\sin \theta }\left|{\vec{F}}_{\text{balloon}}\right|=\frac{r}{2\sin \theta }\left|{\vec{F}}_{\text{balloon}}\right|$$

The absolute value of $\vec{M}$ is given by Eq. (6), and it approximately depends on *r*, *θ*, and $\vec{F}$_balloon_.

#### Formulation of $\vec{{F}}$_balloon_

**Fig. 3:**
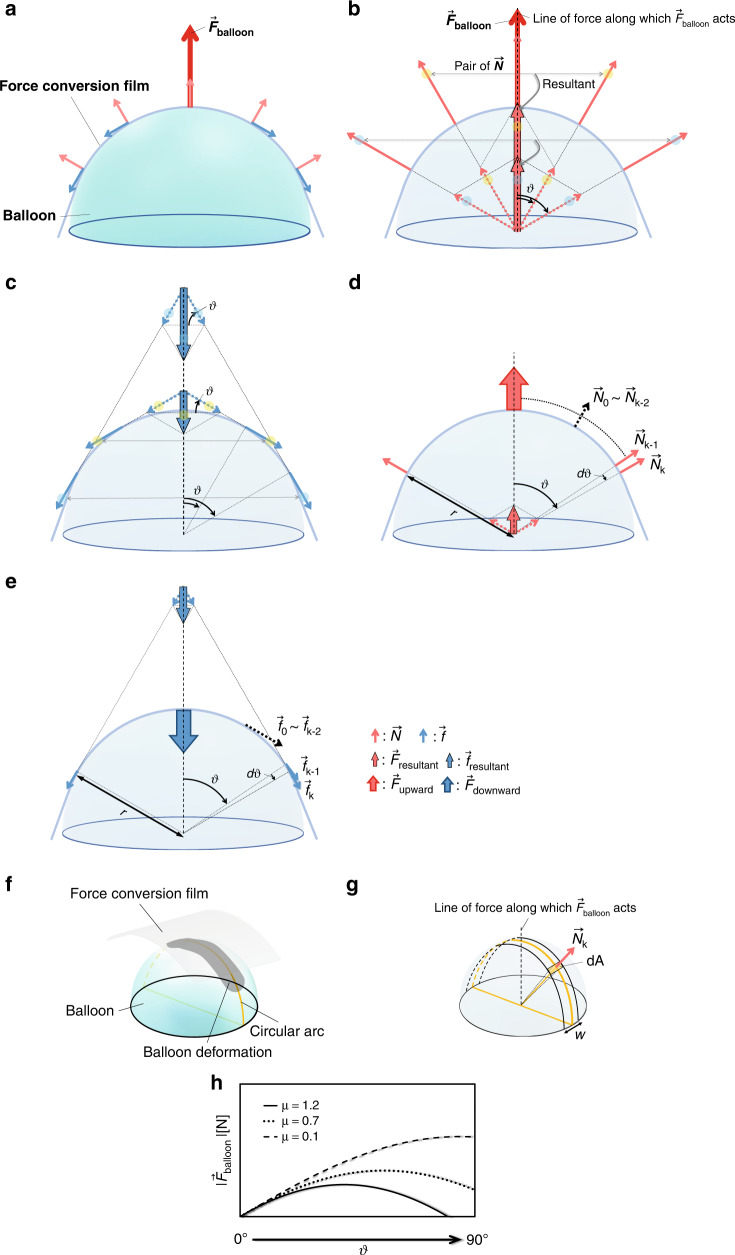
Mechanism of ${\overrightarrow{F}}_{balloon}$ generation. **a** Force occurring between the balloon and the force conversion film: force vector $\vec{N}$ in the normal direction of the balloon surface per unit contact area *dA* and friction force vector $\vec{f}$ generated by $\vec{N}$ as a vertical drag force. **b** The force vector $\vec{F}$_resultant_ from the composition of $\vec{N}$. **c** The force vector $\vec{f}$_resultant_ generated by the composition of $\vec{f}$. **d** $\vec{F}$_upward_, which is the sum of the resultant force ${\vec{F}}_{\text{resultant}(\text{k})}$ at k = 0, 1, …, k arising from ${\vec{N}}_{\text{k}}$ (k = 0, 1,…, k) at each position. **e** $\vec{F}$_downward_, which is the sum of the ${\vec{f}}_{\text{resultant}(\text{k})}$ at k = 0, 1, …, k resulting from ${\vec{f}}_{\text{k}}$ (k = 0, 1,…, k) generated at each position. **f** Characteristics of the contact between the balloon and force conversion film. The contact area occurs at the region of balloon deformation centered on the circular arc that passes through the balloon vertex. **g** The width *w* of the contact area around the circular arc is nearly constant and can be approximated. **h** Change in $\vec{F}$_balloon_ with increasing contact angle *θ* between the balloon and force conversion film: $\vec{F}$_balloon_ increases with decreasing friction coefficient *µ*, and the peak moves to the right (in the direction of increasing *θ*)

Figure 3 shows the mechanism of $\vec{F}$_balloon_ generation. As shown in Fig. 3a, the same force $\vec{N}$ is generated per unit contact area in the normal direction of the balloon surface when the balloon and film contact. The absolute value of $\vec{N}$ is calculated by the following equation:

7 $$\,\left|\vec{N}\right|={PdA}$$

(*P*: applied pressure, *dA*: unit contact area)

As shown in Eq. (7), $\vec{N}$ is an infinitesimal surface element. From Fig. 3b, considering two pairs of $\vec{N}$ forces that are symmetrical with respect to the line of force along which the $\vec{F}$_balloon_ acts, the resultant force acts in the upward direction. Focusing on a pair of $\vec{N}$ forces that exist at an angle *θ* from the line of force along which the $\vec{F}$_balloon_ acts, the resultant force $\vec{F}$_resultant_ generated by the two $\vec{N}$ forces is calculated by the following equation:

8 $${\vec{F}}_{\text{resultant}}\,=2\vec{N}\cos \theta$$

Additionally, Fig. 3a shows the frictional force between the balloon and the force conversion film. The frictional force depends on $\vec{N}$ in Eq. (7), the vertical drag due to the film’s own weight, and the friction coefficient between the balloon and the film. The weight of the film, however, is negligible because it is sufficiently small compared to the expansion force of the balloon. The friction force $\vec{f}$ per unit contact area *dA* between the balloon and the force conversion film is calculated as follows:

9 $$\left|\vec{f}\right|=\mu \left|\vec{N}\right|$$

($\mu$: Friction coefficient between the balloon and film.)

As shown in Fig. 3a, the generated frictional force is vertical relative to $\vec{N}$ and acts in a direction that prevents balloon expansion in the tangential direction of the unit contact area. Figure 3c shows the downward resultant force $\vec{f}$_resultant_ caused by a pair of forces $\vec{f}$ existing at an angle *θ* from the line of force acted on by the $\vec{F}$_balloon_. From Eqs. (8) and (9), $\vec{f}$_resultant_ is calculated as follows:

10 $${\vec{f}}_{\text{resultant}}=2\mu \vec{N}\sin \theta$$

Where values of $\vec{N}$ generated at the position of angle *θ* from the line of force acted on by $\vec{F}$_balloon_ are numbered ${\vec{N}}_{0}$, ${\vec{N}}_{1}$, ${\vec{N}}_{2}$,…, ${\vec{N}}_{\text{k}}$ (k = 0, 1, 2,…, k) starting from the balloon vertex, as shown in Fig. 3d, and the corresponding resultant force ${\vec{F}}_{\text{resultant}(\text{k})}$ (k = 0, 1, 2,…, k) and the sum of these resultant forces, ${\vec{F}}_{\text{upward}}$, are also shown in Fig. 3d. The position k is the contact edge between the balloon and force conversion film. Figure 3e shows the friction force ${\vec{f}}_{\text{k}}$ (k = 0, 1, 2,…, k) generated at an angle *θ* from the line of force along which the $\vec{F}$_balloon_ acts, the corresponding resultant frictional force ${\vec{f}}_{\text{resultant}(\text{k})}$ (k = 0, 1, 2,…, k), and the sum of these resultant forces ${\vec{F}}_{\text{downward}}$. Since the balloon expands isotropically, ${\vec{N}}_{\text{k}}$ is equal at every angle *θ* (all of number k), and the following equation is obtained:

11 $$\left|{\vec{N}}_{\text{k}}\right|=N$$

(k = 0, 1, 2, …, k)

Using Eq. (8), ${\vec{F}}_{\text{resultant}(\text{k})}$ can be obtained as follows:

12 $$\left|{\vec{F}}_{\text{resultant}(\text{k})}\right|=2\left|{\vec{N}}_{\text{k}}\right|\cos \theta$$

Similarly, ${\vec{f}}_{\text{k}}$ (k = 0, 1, 2,…, k) and the corresponding resultant force ${\vec{f}}_{\text{resultant}(\text{k})}$ are as follows:

13 $$\left|{\vec{f}}_{\text{k}}\right|=\mu \left|{\vec{N}}_{\text{k}}\right|$$

14 $$\left|{\vec{f}}_{\text{resultant}(\text{k})}\right|=2\mu \left|{\vec{N}}_{\text{k}}\right|\sin \theta$$

Within the force $\vec{F}$_balloon_ exerted by the balloon on the force conversion film, the upward force that contributes to the generated force is $\vec{F}$_upward_, and the downward force that reduces the generated force is $\vec{F}$_downward_. $\vec{F}$_balloon_ is the sum of $\vec{F}$_upward_ and $\vec{F}$_downward_, and $\vec{F}$_upward_ and $\vec{F}$_downward_ are the sums of ${\vec{F}}_{\text{resultant}(\text{k})}$ and ${\vec{f}}_{\text{resultant}(\text{k})}$ at each k (k = 0, 1, 2,…, k) position. Thus, the following equations hold:

15 $$\left|{\vec{F}}_{\text{balloon}}\right|=\left|{\vec{F}}_{\text{upward}}\right|-\left|{\vec{F}}_{\text{downward}}\right|$$

16 $$\left|{\vec{F}}_{\text{upward}}\right|=\mathop{\sum }\limits_{\text{k}=0}^{\text{k}}\left|{\vec{F}}_{\text{resultant}(\text{k})}\right|$$

17 $$\left|{\vec{F}}_{\text{downward}}\right|=\mathop{\sum }\limits_{\text{k}=0}^{\text{k}}\left|{\vec{f}}_{\text{resultant}(\text{k})}\right|$$

Figures 3d and 3e show that ${\vec{N}}_{\text{k}-1}$ at position k−1 changes angle *dθ* until it reaches ${\vec{N}}_{\text{k}}$ at position k. The following relationship can be obtained at this time using the infinitesimal angle *dθ* from Eqs. (11) and (12).

18 $$\frac{d|{\overrightarrow{F}}_{{\rm{upward}}}|}{d\theta }=|{\overrightarrow{F}}_{{\rm{resultant}}({\rm{k}})}|=2N\,\cos \theta$$

Similarly, from Eqs. (11) and (14), the following equation also holds.

19 $$\frac{d|{\overrightarrow{F}}_{{\rm{downward}}}|}{d\theta }=|{\overrightarrow{f}}_{{\rm{resultant}}({\rm{k}})}|=2\mu N\,\sin \theta$$

Then, to integrate Eqs. (18) and (19), we consider the force per unit contact area $\vec{N}$. Figure 3f shows the contact features of the balloon and the force conversion film. Since the balloon is spherical and the force conversion film is flat, they make contact along the arc that passes through the top of the balloon. The deformed balloon creates a contact area along the arc of contact. In Eq. (7), $\vec{N}$ depends on the applied pressure *P* and the unit contact area *dA*. As shown in Fig. 3g, by approximating the width of the deformed part of the balloon as *w* and the balloon radius as *r*, *dA* is approximated as a rectangle with dimensions given by the arc length of the contact area *rdθ* and the deformed width of the contact area *w*. *dA* can be approximated by the following equation:

20 $$dA=rwd\theta$$

Substituting Eq. (20) into Eq. (7) yields the following equation:

21 $$\left|\vec{N}\right|=N={Prwd}\theta$$

The result of Eq. (21) is substituted into Eq. (18) for integration.

$$\left|{\vec{{F}}}_{\text{upward}}\right|={\int }_{0}^{\theta }2{Prw}\cos \theta d\theta$$

22 $$\left|{\vec{F}}_{\text{upward}}\right|=2{Prw}\sin \theta$$

The absolute value of $\vec{F}$_upward_ is calculated as in Eq. (22), and from Eq. (19), the integral is similarly calculated.

$$\left|{\vec{F}}_{\text{downward}}\right|=2\mu {Prw}{\int }_{0}^{\theta }\sin \theta d\theta$$

23 $$\left|{\vec{F}}_{\text{downward}}\right|=-2\mu {Prw}\cos \theta +2\mu {Prw}$$

Using the aforementioned results, we can calculate the net force in the upward direction, which is the force that is converted into the generated force. $\vec{F}$_balloon_ is the difference between $\vec{F}$_upward_ and $\vec{F}$_downward_, so it is calculated from Eqs. (15), (22), and (23) as follows:

24 $$\begin{array}{l}\left|{\vec{F}}_{\text{balloon}}\right|=2{Prw}\sin \theta -\left(-2\mu {Prw}\cos \theta +2\mu {Prw}\right.\\ \qquad\qquad =2{Prw}(\sin \theta +\mu \cos \theta -\mu )\end{array}$$

On the basis of Eq. (24), it can be confirmed that the absolute value of $\vec{F}$_balloon_ depends on the balloon radius *r*, contact angle *θ*, deformation width of the balloon *w*, and friction coefficient *µ*. Figure 3h is of Eq. (24) visualized with appropriate values substituted. The vertical axis shows the $\vec{F}$_balloon_, and the horizontal axis shows the angle *θ* between the line of force along which the $\vec{F}$_balloon_ acts and the edge of contact between the balloon and force conversion film. From Fig. 3h, the generated force increases with a lower friction coefficient *µ*, and the peak moves in the direction of increasing *θ*. Depending on the value of the friction coefficient, however, $\vec{F}$_balloon_ may decrease with increasing contact angle *θ* between the balloon and the force conversion film.

#### Consideration of balloon radius r and contact angle θ

**Fig. 4:**
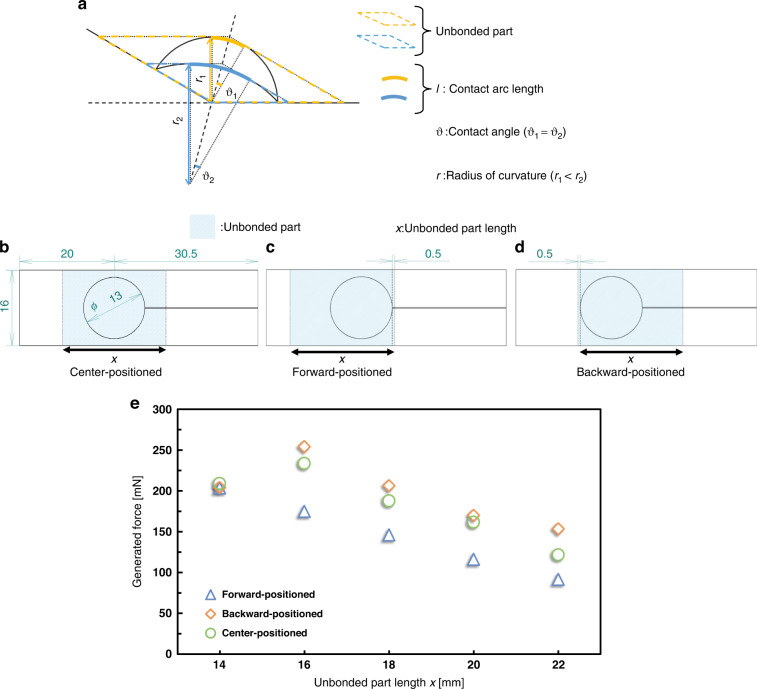
Effect of reducing the length of the unbonded part and changing the bonding position of the force conversion film. **a** As the length of the unbonded part decreases, the radius of curvature *r* increases, and the contact arc length *l* increases. **b** Center-positioned device: The force conversion film was bonded such that the unbonded part was centered on the balloon. **c** Forward-positioned device: The force conversion film was bonded such that the unbonded part was shifted forward against the balloon. **d** Backward-positioned device: The force conversion film was bonded such that the unbonded part was shifted backward against the balloon. **e** Generated force of the designed device shown in Fig. 4b–d with respect to the unbonded part length *x*

From Eq. (6), the generated force of the actuator depends on the balloon radius *r*, the contact angle *θ* between the balloon and the force conversion film, and $\vec{F}$_balloon_. From Eq. (24), $\vec{F}$_balloon_ depends on the balloon radius *r*, the contact angle *θ*, the deformation width of the balloon *w*, and the friction coefficient *µ*. In this section, the balloon radius *r* and contact angle *θ* are also considered. Figure 1a shows a side view of the driving actuator, and Fig. 4a shows a geometrical model based on this side view. The unbonded part forms a parallelogram when the actuator is driven, as shown in Fig. 4a. The arc length *l* of the contact area between the balloon and the force conversion film is calculated using the balloon radius *r* and the contact angle *θ*, as shown in the following equation:

25 $$l=2r\theta$$

As shown in Fig. 3f, the contact area between the balloon and the force conversion film is generated at the region of balloon deformation around the circular arc. Therefore, a larger contact arc length *l* leads to an increasing number of $\vec{N}$, resulting in a larger $\vec{F}$_balloon_. Figure 4a shows the geometric relationship that arises when the bonding position of the force conversion film is changed. It can be seen from the geometric relationship that *θ* is constant even when the bonding position is changed. The balloon radius, however, increases as the unbonded part of the force conversion film (Fig. 4a) decreases in length, and the contact circular arc *l* increases, as calculated in Eq. (25). From Eqs. (6), (24), and (25), it is expected that the actuator produces a higher generated force at a constant *θ* by increasing the balloon radius *r*, increasing the deformation width *w*, and decreasing the friction coefficient *µ*. Equations (6), (24), and (25) demonstrate that an increase in the balloon radius *r* has a significant effect on the generated force. In the following section, the effect of reducing the length of the unbonded part and changing the bonding position of the force conversion film on the generated force of the actuator is discussed.

## Results and discussion

### Characteristic evaluation of devices with a reduced length of the unbonded part

Figure 4b–d shows the design of the device used in this experiment. The unbonded part length *x* of the force conversion film was set to 14, 16, 18, 20, and 22 mm, and the generated force at a device displacement of 10 mm was measured using a load cell. After each measurement, the bonding position of the force conversion film was changed, and measurements were repeated to compare the generated forces with the same device. The PI film was used as the force conversion film, and double-sided tape (NICETACK, NICHIBAN) and adhesive tape (vinyl tape, NICHIBAN) were used to attach the film for easy removal after measurement. As shown in Fig. 4c, d, we also measured the generated forces of the “forward-positioned device” and “backward-positioned device.” These devices have an off-center unbonded part about the balloon, while the conventional devices have a center-positioned unbonded part about the balloon. Figure 4e shows the measurement results. The vertical axis is the generated force, and the horizontal axis is the length of the unbonded part. For all designs, the length of the unbonded part decreased, and the generated force tended to increase as the balloon radius *r* increased (from Eqs. (24) and (25)). The maximum generated force was observed for the backward-positioned device at *x* = 16 mm.

**Fig. 5:**
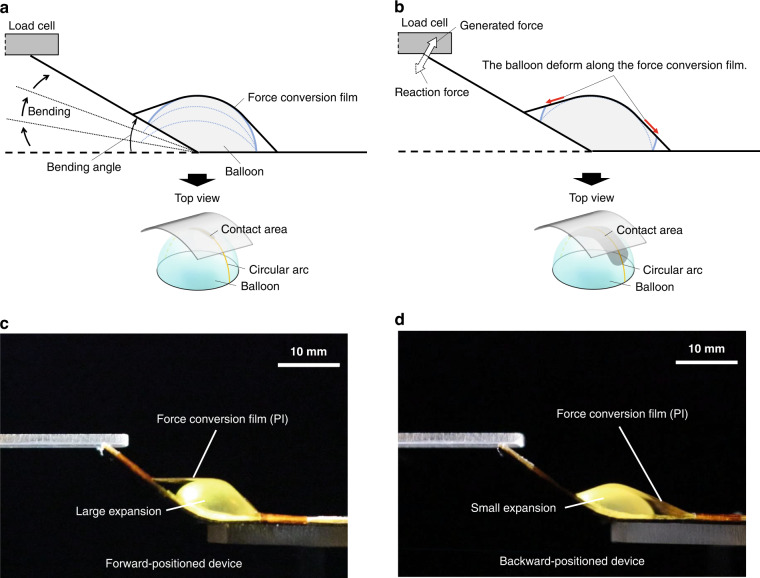
Characteristics of balloon deformation when the device is driven. **a** The bending angle increases as the balloon expands when there is no load at the device tip. **b** The contact area increases when a load is applied at the device tip because the force conversion film is fixed and the balloon deforms along the film. **c** Expansion of the forward-positioned device: The balloon had to expand significantly before tension could be applied to the film in front of it. **d** Expansion of the backward-positioned device: The film in front of the balloon was tensioned even if the balloon expansion was small

The following section describes the factors that led to the maximum generated force for the backward-positioned device and the subsequent reduction in the generated force at *x* = 14 mm. Figure 5a, b show the characteristics of the balloon deformation when the device is driven. The force conversion film lifts as the balloon expands, and the bending angle of the device increases when the tip of the device is unloaded (Fig. 5a). As shown in Fig. 5b, the bending angle of the device becomes constant when a load is applied to its tip, causing the force conversion film to stop moving. As a result, the balloon deforms along the film, while the contact area around the contact arc between the balloon and the force conversion film increases. The amount of deformation of the balloon along the force conversion film at this time determines the deformation width *w* in Eq. (24). In other words, with a smaller balloon expansion when the same load is applied to the tip of the device, the generated force of the designed device should be higher. Figure 5c, d show photographs of the forward- and backward-positioned devices, respectively, when the load was applied to the tip and the tension of the force conversion film began to increase. Figure 5c, d show that the balloon expansion of the backward-positioned device was smaller than that of the forward-positioned device. The smaller the unbonded region in front of the balloon was, the less the balloon expanded when the same load was applied to the device tip, and the wider the deformation of the balloon (denoted as width *w*) was. Consequently, the generated force increased, and as a result, the generated force of the backward-positioned device was maximized, as shown in Fig. 4e.

**Fig. 6:**
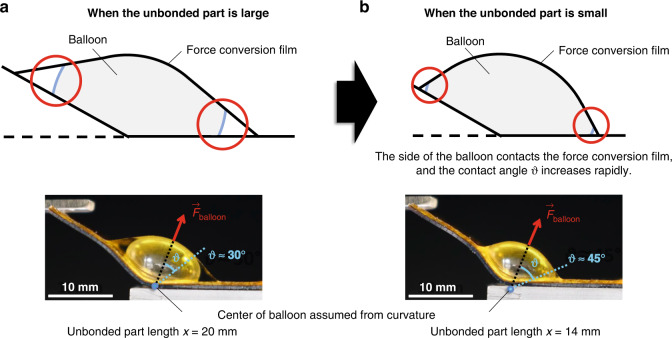
Relationship between the size of the unbonded part and the contact angle. **a** The sides of the balloon do not contact the force conversion film when the unbonded part is large, and the contact angle *θ* is constant. The contact angle *θ* was ~30° when the unbonded part length *x* = 20 mm. **b** The balloon sides make contact with the force conversion film when the unbonded part is small, and the contact angle *θ* increases rapidly. The contact angle *θ* was ~45° when the unbonded part length *x* = 14 mm

Figure 6a, b compare the contact between the sides of the balloon and the force conversion film for different unbonded parts. The contact angle *θ* rapidly increased when the balloon was pressed down by the force conversion film, causing it to make contact with the lower part of the balloon. As shown in Fig. 3h, there were cases in which $\vec{F}$_balloon_ tended to decrease as the contact angle *θ* increased. The decrease in the generated force observed at *x* = 14 mm in Fig. 4e was attributed to the reduction in $\vec{F}$_balloon_ resulting from the effect of friction caused by the increased contact angle *θ*.

From the analysis described above, the optimal design for the friction coefficient *µ* of the currently used materials (PBA and force conversion film) was found to be close to the unbonded length *x* = 16 mm of the backward-positioned device. This was then confirmed experimentally.

### Optimization of force conversion film bonding positions

**Fig. 7:**
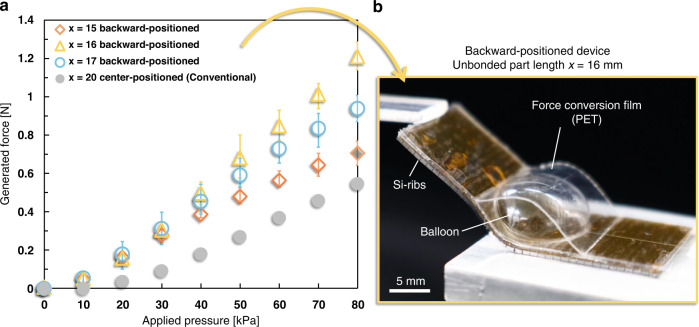
Relationship between generated force and applied pressure. **a** The generated force of the device with unbonded part length *x* = 16 mm was ~2.2 times higher than that of the conventional device (a center-positioned device with *x* = 20 mm). **b** In this experiment, we used PET film (with the improved bonding method) as the force conversion film

In this experiment, we used PET film (with an improved bonding method) as the force conversion film of the device (Fig. 7b) and experimentally derived the optimal bonding position of the force conversion film. Figure 7a shows the relationship between the applied pressure and generated force for unbonded part lengths of *x* = 15, 16, and 17 mm (as shown in Fig. 4d) of the backward-positioned device. The vertical and horizontal axes show the generated force and applied pressure, respectively, indicating the generated force at a 10 mm displacement. Based on Fig. 4e, the optimum design was considered to have an unbonded part length of approximately *x* = 16 mm, so the generated force at *x* = 16 ± 1 mm was measured. The range of applied pressure was 0 to 80 kPa to accommodate the pressure endurance value of the balloon. For comparison, the generated force of the conventional device is also shown in Fig. 7a. The conventional device was the center-positioned device with *x* = 20 mm. As shown in Fig. 7a, the device with an unbonded part length of *x* = 16 mm had the largest generated force, which was 2.2 times (80 kPa) higher than that of the conventional device.

#### Effects of frictional force

As depicted in Fig. 4e, a decrease in the generated force was observed at an unbonded part length of *x* = 14 mm (center and backward-positioned). As *x* decreases, the contact angle *θ* increases, as shown in Fig. 6, and the generated force is expected to decrease, as shown in Fig. 3h (high µ). Figure 3h shows that a lower friction coefficient *µ* results in a higher generated force, and under this condition, the maximum generated force was expected at *x* = 14 mm, where *θ* was the largest. Since the results in Fig. 3h were derived based on the center-positioned device model, the generated force characteristics of lower friction for the center-positioned device were examined to determine whether the tendencies of the force characteristics aligned with the theory, as shown in Fig. 3h.

**Fig. 8:**
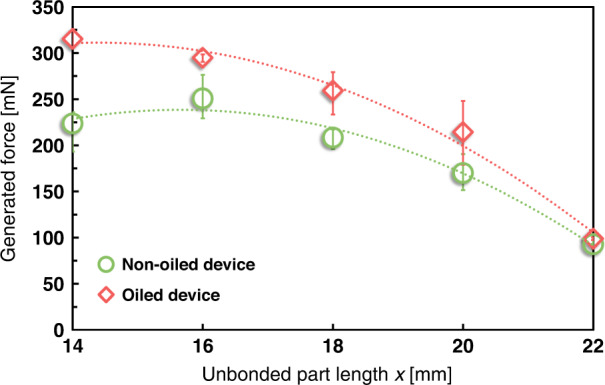
Effect on the generated force of reducing the friction coefficient by applying oil. Dotted lines represent the polynomial approximations. The generated force increased because the oil reduced the friction coefficient. The rate of increase was higher for a smaller unbonded part length *x* (larger contact angle *θ*), showing the same characteristics as in Fig. 3h

Figure 8 compares the generated force for center-positioned devices with unbonded part lengths *x* = 14, 16, 18, 20, and 22 mm when the friction coefficient was reduced. Oil was applied to decrease the friction coefficient, and the generated force was measured under the same conditions used in the experiment, as shown in Fig. 4e. The friction coefficient of the oiled device was ~0.1 and that of the non-oiled device was ~1.2. As shown in Fig. 8, the generated force was larger for the oiled device with reduced friction, and it reached the largest at *x* = 14 mm, when the contact angle *θ* was at its maximum. These results were consistent with the determined tendencies presented in Fig. 3h.

On the basis of the aforementioned findings, we expect to achieve even higher outputs by using materials with lower friction coefficients to construct the actuator and by applying lubricants.

## Conclusions

This paper presents the design of an inflatable bending actuator with a high force generation efficiency that uses a conversion mechanism based on a mechanical model. In this actuator, the balloon is expanded by applying pressure, and the force conversion mechanism converts the expansion force into a bending force to drive the actuator.

We focused on the contact area between the balloon and the force conversion film, which occurs at the deformed part of the balloon centered on the arc of contact, and we aimed to increase either the arc length of the contact arc (*l*) or the balloon deformation width (*w*). As a result, we proposed reducing the length of the unbonded part to achieve a high-output and efficient design. The results showed that the generated force initially increased as the length of the unbonded part decreased but subsequently decreased when the length of the unbonded part was further reduced. The generated force for the backward-positioned device was the largest. The results confirmed that for the friction coefficients of the currently used materials, the backward-positioned device was optimal, and, compared to that generated by the conventional design, this device successfully increased the generated force by ~2.2 times. Oil was applied between the balloon and the force conversion film to confirm the effect of the friction force, and the generated force was evaluated. The results indicated that the tendencies observed in the theoretical models were consistent with the experimental results. Therefore, it is expected that additional increases in output could be achieved through further decreases in the friction coefficient.

## Materials and methods

### Fabrication process of the inflatable film actuator with bending motion

Figure 1a shows the appearance of the inflatable film actuator, and Fig. 1c shows its structure. As shown in Fig. 1c, this actuator consists of a balloon structure (PBA) with a force conversion film on the surface and a 25-μm-thick polyimide film and 400-μm-thick Si-ribs on the back^16,17^. The PBA was fabricated using silicone rubber (KE-1606, Shin-Etsu Chemical Co., Ltd.). In our previous study, the force conversion film was made of polyimide film; however, in this study, a 25-μm-thick polyethylene terephthalate film (PET: Lumirror T60, Toray Industries, Inc.) and KE-1606 were used to improve the bonding performance. Since the force conversion film converts the expansion force of the balloon into a bending force, a superior bonding force with the PBA improves the force conversion efficiency between the expansion and bending forces. This is because by reducing the separation of the bonding surfaces, the tensile force of the force conversion film is transmitted to the acting part without decreasing. As a result, the generated force has improved.

PET films were bonded to KE-1606 thin films (KE-1606 was spin-coated at 2000 rpm on a Si wafer and thermally cured at 85 °C for 10 min) using a silane coupling agent (3-mercaptopropyltrimethoxysilane) and irradiated with ${\text{O}}_{2}$ plasma. The PBA was then bonded to KE-1606, which was bonded to the PET film surface using surface activation (vacuum ultraviolet light).

### Experimental setup

As shown in Fig. 1a, in this experiment, the generated force of the device at a displacement of 10 mm was measured for comparison with that of devices from previous works by combining a load cell (LVS-500GA, Kyowa Electronic Instruments Co., Ltd.) and a strain amplifier (DPM-911B, Kyowa Electronic Instruments Co., Ltd.). The device was fixed to a Z stage (TBM-603, SIGMAKOKI), and the positioning of 10 mm displacement was controlled by a gauge equipped with the Z stage. The device was driven by applying pressure with a syringe pump (PUMP33, Harvard Apparatus). The pressure applied to the device was measured with a manometer (PG-100, Nidec). To reduce the frictional force, we applied a thin layer of silicone oil (KF-96-50CS, Shin-Etsu Chemical Co., Ltd.) between the force conversion film and the balloon in certain experiments. The friction coefficient was measured by contacting objects made of the same material as the force conversion film and balloon and then measuring the angle at which the object began to slide when tilted.
